# Cases of Metastasis, or Translation of Diseased Action

**Published:** 1810-09

**Authors:** 

**Affiliations:** Surgeon


### Cases of Metastasis, or Translation of diseased Action. By Mr. Howship, Surgeon.

### *A Case of translated diseased Action, producing Death by Suffocation.*

In the year 1801, a soldier was admitted into the General Military Hospital at Mahon, in the island of Minorca. His complaint was a very hot, red, and painful tumour upon the muscular part of the left thigh. The tumour was hard, and excessively sensible, and had very much the characters of phlegmonoid inflammation. From the very painful state of this swelling, and the constitutional sympathy attending it, his rest at night was almost totally prevented.

This swelling, by the assistance of cold and sedative applications to the part, laxative and cooling medicines, and a spare diet, was nearly disperse within a week. By the time the swelling on the thigh had almost entirely disapdisappeared, a second tumour, having precisely the same characters as the first, arose upon the fleshy part of the left fore arm. This swelling was productive of the most severe pain. It was poulticed and fomented for three days, which treatment materially assisted its maturation. The abscess being opened, about four ounces of matter were discharged. This wound was situated just upon the flexors of the hand and fingers, and continued to discharge very copiously for a week.

About the same time, a very violent degree of inflammation, heat, swelling, pain, and tension, arose upon the back part of the right hand. This was fomented and poulticed three days, during which time he had not the least power of extending or even moving his fingers. On the fourth day from its appearance the symptoms began to abate, and by the end of the week it was entirely dispersed.

During the period of the ulceration on the left fore arm, several fragments of tendons, in a sloughy state, appeared about the edges of the sore, and these were from time to time cut off.

Judging principally from the large quantity of matter, it was considered that this ulcer might run some distance down among the tendons of the hand; and on examination, this proved to be the case, for a probe found no interruption in passing down its whole length towards the wrist. A sinus was also found, extending upward toward the elbow; and near the end of the week, the cavity of the ulcer was much disposed to throw up a fungous mass. This ulcer continued to discharge for about ten days, and then the quantity of matter formed, on a sudden diminished. In two days more the sore was absolutely dried up, and the applications were found not in the least degree soiled by discharge of any kind. From that day he felt something unusual in his throat. This sensation in the throat he complained of a few days afterward, being under some alarm. At this time respiration was impeded, not in such manner as to lead to any suspicion of oppression or complaint in the chest, but such impediment as arises from any obstruction in the trachea, preventing the free passage of the air. In the attempt to speak, it appeared that he was only able to articulate very partially; and it seemed that something of mischief was going on in the larynx.

Towards evening this impediment amounted to the most alarming and distressing difficulty; the countenance ex-

 pressed pressed that sort of wild despair which attends the unsuccessful endeavour to keep up the proper actions of the chest. In the night this difficulty of breathing was aggravated to a most astonishing degree; the respiration was extremely laborious and noisy, and he was only able to pronounce what he said in a whisper.

From the constant wheezing noise appearing to arise in the cavity of the larynx, it was conjectured that it might be the consequence of a lodgment of viscid mucus obstructing the passage of the air, yet it was very difficult to conceive that mucus could be so consistent as to produce the regular, clear, even toned sound heard in this case.

The distress having now arrived at its highest pitch, the poor man was raised and supported in bed by the assistance of pillows placed behind his back and shoulders, with a view to relieve his breathing. This expedient, however, did not seem to be attended by any benefit whatever. At midnight a consultation was called, for the patient was evidently in the most extreme danger of suffocation. It was with very little confidence that any sort of treatment was proposed; it was indeed suggested, that the complaint might be something of spasmodic action; and upon this conjecture, opiates, ætherials, &c. were repeatedly given. Added to these medicines, a large blister was laid below the sternum. Nothing that was done, apparently, produced the least alteration, nor the smallest alleviation in the symptoms. About six in the morning he expired, completely exhausted by the increasing urgency of the dyspnœa.

### Examination after Death.

The integuments covering the neck being divided, the trachea, larynx, &c. were removed. There was no appearance of any morbid or excessive secretion of mucus, either about the cavities of the larynx, or within the trachea. The whole of the mischief in these parts was confined to the larynx, the cavity of which was so altered in its form by disease as to have evidently been the cause of suffocation.

When the parts were clean washed and carefully examined, it was ascertained that the inner membrane of the larynx had been in a state of very acute inflammation. The inflamed mucous membrane within the larynx was greatly increased in thickness. That portion of the membrane lining the inside of the thyroide cartilage below the arytenoide cartilages was astonishingly thickened, constituting tuting on both sides of the cavity a spongy elastic sort of cushion, projecting into the cavity in such manner that the two opposite surfaces were found nearly as possible in perfect contact. This must have proved the most formidable impediment to the transmission of the air.

These portions of the diseased membrane had a firm, fleshy, red appearance, and on being cut into and the divided parts pressed, globules of thick purulent matter issued forth very numerously from this diseased structure.

Viewing the entire larynx from above, it was obvious enough that another material cause for the fatal event of the case existed in the very straitened passage between the two arytenoide cartilages. The universal thickening of the laryngal membrane not only had the effect of pressing the two cartilages almost into contact with each other, but had also still more contributed to close up the channel by increasing the quantity of solid matter covering the cartilages themselves.

---

*A Case, in which there was a Translation of diseased Action from the Surface of the Body to the Brain, or its Membranes, terminating fatally.*

G. Dunscombe, a strong, healthy, labouring man, 50 years of age, was engaged as a farmer's servant in the country. In October 1809, the weather at the time being warm, he had, very imprudently, drank repeatedly and heartily, both of cold water and table beer, while heated by hard work, and while a very copious perspiration was breaking forth. Soon afterward he found himself ill and feverish. This indisposition continued, and in a few days a rash came out all over his body and limbs, and still he burned like fire to the sensation of others, while to his own feelings, it seemed that he was shivering with excessive cold.

When he became so ill that he could no longer move about, medical advice was procured, and he was several times immersed in the warm bath. The rash after remaining upon the skin for some days, disappeared; within a week it came out again. In this manner it returned upon him several times.

About six weeks after he first found himself ill, the rash having finally subsided, he complained of a great soreness and pain that was arising in one of his groins, this was attended with heat, redness and tumour. There were also other swellings beneath the integuments, upon various parts of the thigh and arm. Some of these, assist-

 ed ed by poultices and fomentations, formed collections of matter; these were let out in a week after the commencement of the inflammation. Others were less disposed to suppurate, and remained in a comparatively indolent state. In this kind of ill health he remained confined till the January following, when after having passed a tedious course of pain and distress, it seemed that they were in general disposed to subside and dry up. The swellings not advanced to suppuration, tending to resolution, while those that had ulcerated, furnished less and less discharge daily.

Just at the time when these ulcers ceased to discharge at all, the man was observed to be more than usually disposed to doze, or rather sleep. This happened on January 15, from which period the disposition to sleep increased upon him daily. He became morose and impatient. When it was observed to him that he had become very sleepy of late, he said, No, he was not sleeping as often as his eyes were shut, but that he could hear every thing, and see every thing as well other people.

This propensity to slumber continued to increase upon him, although by slow degrees. On the 21st, he felt a presentiment of his situation. He said to his wife, who frequently visited him, that he was convinced he should not live long. He complained of no pain either in his head or elsewhere; on the contrary, he was so well as to be able to sit up in a chair while his bed was made. It is remarkable, that while he was sitting up, he observed, that something ailed his eyes, for that he saw every thing double. In the night following he was seized with a fit of convulsive catchings in all the voluntary muscles. This attack lasted for a few minutes only, and then subsided.

The morning after this, that of the 22d day of the month, he was found laying in a state of insensibility, breathing laboriously. In the forenoon he had a second attack of mild convulsive motions in the voluntary muscles. A third fit occurred at eleven in the forenoon, and at three in the afternoon he expired.

The pulse in the early part of this man's illness had been febrile and hard, but latterly, it had been constantly weak, small, and slow.

Circumstances, unfortunately, prevented the examination which should have been instituted after this man's death.

---

*A Case*

*A Case of translated morbid Action, in which, from the sudden Suppression of an habitual Discharge by Perspiration, Symptoms indicating Effusion within the Head took place.*

J. Powell, a very healthy old man, aged 77, had been for many years subject to an excessive degree of perspiration from the feet, more especially after taking any exercise. This tendency to sweating in the feet had for years past so great an inconvenience, as to oblige him sometimes to change his stockings several times in the course of the same day.

In all other respects, he enjoyed very good health, and his constitutional strength was remarkably great for a person of his advanced age.

Complaining frequently of this inconvenience, he was one day advised by a neighbour, to apply the fresh leaves of dock to his feet; he was told that this would effectually cure his complaint. Accordingly he laid a single dock leaf to the sole of each foot, and very soon perceived they had taken effect. He felt a considerable sensation of tingling and irritation wherever the leaves came in contact with the skin. Within half an hour after they were applied, he felt great uneasiness and pain in his head. This pain soon became very bad, particularly over the eyes, which were so much affected, that before the leaves had been applied an hour, he was very nearly totally deprived of his sight. His sight became affected when the pain was merely an uneasiness; and the power of distinguishing the objects around him was completely gone within the hour. Upon being examined with regard to his power of discerning the light, it appeared that he could perceive a strong light, and he could also make out the figure of any object placed immediately between a strong light and his own eye. In such circumstances, the objects before him seemed as if they were enveloped in a thick fog or mist.

In this state he continued to remain for some time; during the following night, the pain in the head was so severe, as to deprive him of his rest; but he had still no constitutional disturbance, nor tenden y to fever.

The next day he was found to be precisely in the same state as before he went to bed. The pupils of the eyes did not shew any disposition to vary or change. They were exposed to different degrees of light, but they remained unaltered, and were fixed in a state of permanent contraction. He could, however, still perceive when he was

 brought brought near a window, but this was all that he could discern.

For his relief, a blister was recommended to be laid behind each ear, and others to be applied on each foot, to be laid between the inner ancle and sole of the foot; small doses of calomel also were directed to be frequently given, as it was intended that his system should be brought under the influence of the mercurial excitement.

As soon as the blisters began to operate and become painful, so soon he perceived the pain in the head and the affection of sight relieved. By the time they were dressed the day after, he found his situation so far improved, that he was able to distinguish many objects with tolerable precision, that before were totally invisible. The blistering plasters removed, dressings of an irritating nature were applied, as it was deemed necessary to keep up a considerable discharge for some time. In addition to the above, it was also directed that his feet should be immersed in warm water morning and evening, and afterward very warmly wrapped in flannels, to produce and encourage a freedom of perspiration.

Under a continuation of this treatment, the patient was gradually restored to health, losing the distressing pain in his head, while he found his sight returning to him more perfectly every day.

The mercurial course affected his mouth rather smartly, and under its influence, he had the comfort of finding himself released from the little remaining head-ach, and very nearly the whole imperfection of vision.

Previous to his illness, he had enjoyed a clearness of sight very unusual at this age; and after his restoration, his vision was very nearly, but not quite, equal to what it was before his confinement.

He was recommended to wear a piece of oiled silk wrapped round each foot, to promote the insensible perspiration.

*Mill Street, Hanover Square,*

*August* 13, 1810.

ACCOUNT

